# The Effect of an Authentic Acute Physical Education Session of Dance on Elementary Students' Selective Attention

**DOI:** 10.1155/2018/8790283

**Published:** 2018-02-05

**Authors:** P. H. Kulinna, M. Stylianou, B. Dyson, D. Banville, C. Dryden, R. Colby

**Affiliations:** ^1^Arizona State University, Tempe, AZ, USA; ^2^The University of Queensland, Brisbane, QLD, Australia; ^3^University of Auckland, Auckland, New Zealand; ^4^George Mason University, Fairfax, VA, USA

## Abstract

There have been calls to test the potential benefits of different forms of physical activity (PA) to executive function, particularly in authentic settings. Hence, the purpose of this study was to investigate the effect of an acute dance session within an existing physical education class on students' selective attention. The study employed a pre/posttest quasi-experimental design with a comparison group in one Aotearoa, New Zealand, primary school. Participants were 192 students (comparison group = 104 students) in Years 5 and 6. The intervention group participated in a dance-based physical education lesson while the comparison group continued their regular classroom work. PA during the physical education lesson was monitored using accelerometers. Selective attention was assessed at pretest and after the comparison/physical education sessions with the d2 Test of Attention. 2 × 2 ANOVA results suggested a significant time effect for all three measures, no significant group effects for any measures, and significant time by group interactions for TN and CP but not for *E*%. The intervention group improved significantly more than the comparison group for TN and CP. This study's findings suggest that existing school opportunities focused on cognitively engaging PA, such as dance, can improve aspects of students' selective attention.

## 1. Introduction

Beyond the well-established health benefits of physical activity (PA) engagement for children and adolescents (e.g., [1]), in the last decade there has been an increased emphasis placed on exploring the relationship between PA and learning-related outcomes in the specific population. Indeed, several review studies suggest that acute bouts of PA can positively influence children's cognitive and academic performance [2–4]. For example, during the 2016 Copenhagen Consensus Conference, a group of researchers from various countries concluded that “a single session of moderate PA has an acute benefit to brain function, cognition, and scholastic performance in children and youth” [2, p. 1]. Equally significant is the conclusion by many studies that time invested in PA at school does not negatively impact children and youth's academic performance [2, 4].

An aspect of cognitive functioning that has received considerable attention in this area is that of executive functioning (e.g., [5–7]). Executive functioning, which is often also called executive or cognitive control, involves cognitive processes responsible for organizing and controlling goal-directed behavior, which are thought to be essential for success in school and life in general. Relevant literature suggests the existence of three core executive functions [5, 6, 8]: (a) working memory, also called updating (holding information in mind and updating existing information with newer, more relevant information), (b) inhibition or inhibitory control (ability to control attention, behavior, etc. and focus on a given/appropriate task by overriding or resisting internal or external impulses, temptations, or distractor interference), and (c) cognitive flexibility, also called shifting (moving between tasks or adjusting to changed demands, circumstances, or priorities). Executive function processes have been examined relative to different types of PA interventions, both acute and chronic, in various child populations [5, 7, 9–11].

Several studies in the body of literature examining PA and executive functions focused in particular on the effects of acute bouts of PA on various aspects of inhibition or inhibitory control, including various types of attention, with promising results [11]. These studies employed interventions in various settings, including the classroom/school (e.g., [12–14]) and the laboratory (e.g., [15]). They also investigated the effect of various PA characteristics, including duration, intensity, and type of PA. Increasingly, however, there is more emphasis placed on the qualitative characteristics of PA that might influence children's cognitive functioning [16, 17] and there have been calls to test the potential benefits of different forms of PA on executive function [16].

Studies that have examined the impact of qualitatively different types of PA generated mixed findings. For instance, some studies reported improvements in attention following acute bouts of aerobic PA with no particular cognitive demands (e.g., [12, 18, 19]). The findings of other studies in children and adolescents suggest that cognitive demanding PA is more beneficial for attentional performance [13, 20, 21], although similar studies in this area reported no performance differences with tasks of differing cognitive demands [22]. Despite these mixed findings, there is consensus among scholars in the field that cognitive or mental engagement during PA [13, 17] or “moving with thought” [16], which can be defined as behavior requiring high cognitive effort or challenge, is a qualitative characteristic that needs to be further explored in future relevant studies.

This study aimed to address the need for further research into the qualitative characteristics of PA that might influence children's cognitive functioning [16, 17] and calls to test the potential benefits of different forms of cognitively engaging PA, such as dance, for executive function [16]. Further, this study aimed to respond to calls for more relevant research in authentic real-world settings [22], as the majority of studies that have examined the impact of acute bouts of PA on executive functioning of children and youth focused on interventions rather than authentic, regularly scheduled PA sessions already taking place in schools, thus lacking ecological validity. As such, the purpose of this study was to investigate the effect of an existing acute session of dance activities within a physical education class on students' selective attention. Selective attention, the ability to “select and focus on particular input for further processing, while simultaneously suppressing irrelevant or distracting information” [23, p. 30], is a component of inhibition or inhibitory control, one of the three core executive functions. This cognitive process is important to be explored in school settings as it positively contributes to learning and academic performance [23]. The hypothesis for this study was that the selective attention of students participating in an acute session of dance activity within a physical education class would be significantly improved compared to the performance of a comparison group.

## 2. Method

### 2.1. Participants and Context

Two hundred and twenty students initially returned parental permission forms to participate in the project. After teachers were randomly assigned to conditions, there were 109 students in the comparison group and 111 students in the intervention group. Fourteen students were absent at either pretest or posttest, and 14 were excluded for low participation levels in physical education. Thus, the final sample included 192 students, 104 students in the comparison group and 88 students in the intervention group (girls = 97, boys = 95; Year 5 = 92 students, Year 6 = 100 students). Participating students were recruited from six Year 5 and Year 6 classes (equivalent to Grades 4 and 5 in the US) in one primary school (elementary school) in Aotearoa, New Zealand (NZ), that primarily serves high socioeconomic status families. Students ranged from age 8 to age 11 (*M* = 9.5 years, SD = .54). At the time of the study, the school had a total enrolment of 645 students with 56% NZ European, 25% Chinese, 11% Indian, and 8% Maori ethnicities.

The eight teachers involved in this study were generalist classroom teachers (six female; two male). One teacher was from Vietnam and the other seven teachers were NZ European. Participating teachers had five to 17 years of teaching experience (*M* = 11.05 years; SD = 7.41). These generalist classroom teachers were responsible for teaching eight different subject learning areas, including physical education, which is typical in the NZ elementary school system. To gain teacher registration as a generalist teacher, these teachers had only been required to take one physical education course (18 contact hours) during their teacher preparation.

Several PA opportunities were available at the primary school where the study took place. These opportunities included a 20-minute morning break (i.e., “morning tea”) and a 60-minute lunch break, as well as two 45-minute physical education lessons per week. Most of the students walked to school as active commuting is common in NZ school communities.

### 2.2. Research Design

This study employed a pre/posttest quasi-experimental design with a comparison group. Eight Year 5 and Year 6 classrooms were randomly assigned into the comparison and intervention groups (using a random number selector in SPSS). The intervention group participated in a physical education lesson while the comparison group engaged in regular classroom work (the two conditions are described in more detail below). The dependent variable was selective attention measured through the d2 Test of Attention [24, 25]. PA levels during the physical education lesson were measured using the New Lifestyles NL-1000 accelerometer. Accelerometer training, d2 Test training, pretest data collection, intervention, and posttest data collection took place over two weeks at the school.

### 2.3. Conditions

The intervention used in this study involved participating in a 45-minute physical education lesson offered at the school as part of students' regular schedule. The physical education lesson was facilitated by each classroom teacher and was based on a popular program in NZ called JUMP JAM (http://www.JUMPJAM.co.nz). JUMP JAM is an aerobic program that combines dance and fitness and was developed for students at the primary and intermediate levels (i.e., elementary and middle school). It includes a number of routines designed to challenge fundamental movement skills, increase fitness, develop student leadership, and motivate students to move and enjoy exercise using contemporary music and movements. During the physical education lesson, a JUMP JAM video was used or live students led a prescribed JUMP JAM routine that was selected by the students.

While the intervention group participated in the physical education lesson, the comparison group continued their regular morning school work in the classroom. This was nonactive and consisted of reading and/or writing tasks.

### 2.4. Protocol

Prior to commencement of this study, the study was approved by the Institutional Review Board of the University of Auckland and the principal of the participating school. Following school approval, the teachers and students of the six Year 5 and 6 classes were invited to participate in the study. Prior to data collection, teachers provided their consent, and parental consent and student assent forms were also obtained.

Prior to data collection, participants practiced wearing the accelerometers and completing the d2 Test of Attention. Practicing with the d2 Test of Attention involved research team members explaining the test to the participants, completing a few lines, and providing time for questions. Subsequently, all participating classes participated in the pretest of the d2 Test of Attention. This took place over several days following morning recess (20 minutes) and morning lessons (120 minutes). Morning recess included having students eat their snack and then either read, play outside, or chat with friends. There was minimal to no activity during lessons in the classroom as students were setting at their desk listening to their teacher or engaged in personal or small group work.

**Figure 1:**
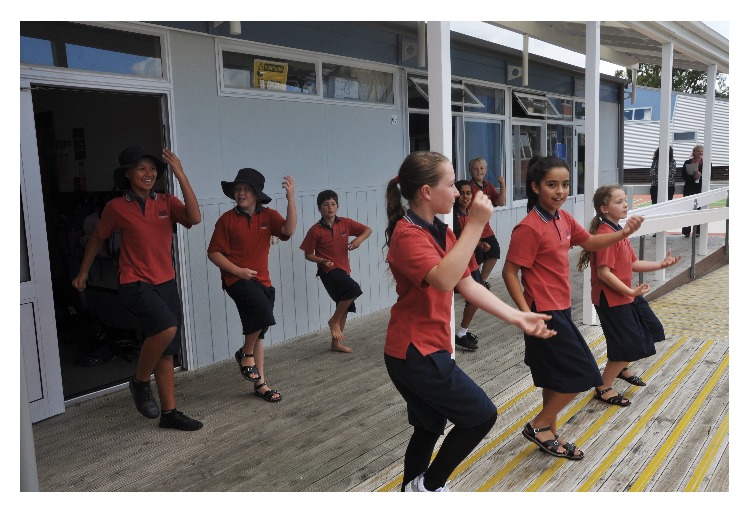
Year six students leading a JUMP JAM physical education lesson.

On posttest days, both groups of students (intervention and comparison) had a morning recess of 20 minutes early in the day and then morning lessons in the classroom (120 minutes). Intervention group participants followed these activities with the 45-minute physical education lesson while wearing an accelerometer to measure their level of PA (see Figure 1). Students put their accelerometers on right before the physical education lesson began. At the initiation of the 45-minute class, students walked to the activity area (5 minutes), engaged into the JUMP JAM activities for their lesson (35 minutes), then walked back to their classrooms (5 minutes), put their accelerometer on the counter, and returned to their seats. Thus, students walked for 10 minutes and participated in JUMP JAM activities for 35 minutes of their 45-minute physical education lesson. During the same time, students in the comparison group continued their regular, nonactive classroom work.

The posttest of the d2 Test of Attention was given to students in the intervention group immediately following their physical education lesson. Students in the comparison group completed the posttest of the d2 Test of Attention at approximately the same time (within a 15-minute timespan) since five of the research team members (all present every day) first gave the test to students in the intervention group in their classrooms and then gave it to students in the comparison group in their classrooms.

The administration of the d2 Test of Attention followed the exact protocol from the test administration guidelines [24]. The instructions from the manual were typed out and read by a research team member every time the test was given.

## 3. Measures and Instruments

### 3.1. Selective Attention

The d2 Test of Attention assesses an individual's ability to focus on a single stimulus, while simultaneously suppressing knowledge of competing distractors. The cognitive procedure of selective attention is needed to successfully complete the test. The specific test is a cancellation test completed with paper and a pencil. The test is comprised of 14 lines of randomly mixed “d” or “p” letters (47 letters in each line). Participants are asked to cross off the letter “d” across each line but only when there is a pair of dashes either above or below the “d” or individually above and below the “d,” while suppressing competing distractions (e.g., “p” and “d” with one, three, or four dashes). During the administration of the test, an acoustic signal is used for participants to move onto the next line after 20 seconds. The entire test is completed in 4 minutes and 40 seconds.

According to recommendations by test developers [24], three measures were calculated and used as indicators of selective attention in this study. The first measure is the total number of items processed (TN), a quantitative measure of processing speed, which is calculated as the sum of the total number of items processed across the test (14 lines). The final letter crossed out on each line (whether correct or not correct) is considered the final item processed in the specific line. The second measure is the percentage of errors (*E*%), a qualitative measure of accuracy and carefulness, which is calculated as the ratio of the number of errors to the total number of items processed. The total number of errors (TE) is calculated by summing up errors of omission (d2 target items processed but not crossed out) and errors of commission (distractor items that were processed and crossed out). The third measure is concentration performance (CP), which is the number of correctly crossed out relevant items minus the errors of commission. While TN-TE is another indicator of overall performance (in addition to CP), Brickenkamp [24] recommends using CP as it provides an “excellent index of the coordination of speed and accuracy of performance,” which, in contrast to TN-TE, “cannot be distorted by such tendencies as the haphazard skipping-over of sections of the test lines, or the crossing out of all letters without discriminating among them” (p. 11). Further, we did not use fluctuation rate (i.e., the difference of the line/lines with the maximum and minimum number of items processed) because it is one of the less reliable measures of the test [24].

Test developers [24] have reported that performance on the d2 Test of Attention is not correlated with an individual's IQ. Rather, it provides information about the speed at which an individual performs visual perception as well as their concentration capacities. Available data indicate that the test has good psychological and construct validity as well as high internal consistency reliability across parameters [24].

### 3.2. Physical Activity

PA during physical education classes in this study was monitored using the New Lifestyles NL-1000 accelerometer. This instrument uses a sampling interval of 4 seconds (i.e., detects the maximum acceleration over each 4-second epoch), which is suitable considering children's sporadic PA patterns. Subsequently, each epoch is categorized into 1 of 11 intensity levels. In this study, moderate-to-vigorous PA was calculated using the 4–9 intensity range, which based on previous research corresponds to an estimated 3.6 MET threshold for moderate intensity [26]. A validation study in a similar population of children (i.e., students in Grades 5 and 6 in the US) showed that the instrument produces an accurate measure of steps and time spent in moderate-to-vigorous PA [27].

Prior to using the accelerometers, batteries were replaced and shake tests performed. All students practiced wearing the accelerometers in the classroom prior to the study (with a member of the research team present to help). Accelerometer data were entered immediately so that any unusual numbers could be checked with participants. Accelerometers were put on prior to leaving the classroom and were removed immediately upon returning in the classroom.

### 3.3. Data Analyses

All analyses were conducted using the Statistical Package for the Social Sciences (SPSS, Version 24.0). Descriptive statistics were calculated for all variables including the steps taken and time spent in moderate-to-vigorous PA (MVPA). According to Brickenkamp [24], no gender differences have been observed in d2 Test of Attention results, so gender differences were not explored in the current study. Fourteen students were excluded from the final analyses due to <5 minutes of MVPA (mean of 3.3 minutes) to ensure a minimal level of engagement in physical education.

Data analyses included 2 (pretest, posttest) × 2 (comparison, intervention group) mixed factor ANOVA tests for the three selective attention measures (TN = total number of items processed; *E*% = errors; CP = concentration performance), followed by an independent samples *t*-test using difference scores (posttest score – pretest score). These tests were run separately for the three selective attention measures. To quantify the magnitude of differences, effect sizes for the 2 × 2 mixed factor ANOVA were calculated using partial eta-squared (partial *η*^2^), for which sizes of .01, .06, and .14 signify small, medium, and large effects, respectively [28]. Cohen's *d* was calculated for the independent samples *t*-test, for which sizes of .2, .5, and .8 signify small, medium, and large effects, respectively [28].

## 4. Results

During their physical education lesson, the intervention group students accumulated, on average, 1931 steps (SD = 421) and 8 : 81 (SD = 2 : 22) minutes of MVPA. This means that students spent about 20% of the total scheduled lesson time (i.e., 45 minutes) or 25% of the actual lesson time (i.e., 35 minutes, excluding transition time from/to the classroom) in MVPA.

Table 1 presents the results of the 2 × 2 mixed factor ANOVA for all three measures of the d2 Test of Attention (TN, *E*%, and CP).  Table 2 presents descriptive statistics for all three measures of the d2 Test of Attention (TN, *E*%, and CP) for both groups, including pretest, posttest, and difference scores. As can be observed in Table 1, 2 (pretest, posttest) × 2 (comparison, intervention) ANOVA results showed no significant effect of group for any measures. However, ANOVA results showed a significant effect of time (pretest, posttest) for all three measures, which indicates that participants across the board improved their performance in all three measures of the d2 Test of Attention from pretest to posttest. ANOVA results also showed a significant group × time interaction for TN and CP, but not for *E*%. The significant interactions indicate different progressions from pretest to posttest for the two groups. Significant interactions were followed up by independent *t*-tests. Results using difference scores showed that the intervention group had improved significantly more than the comparison group from pretest to posttest for both TN [*t*(190) = 2.03, *p* = .04, and *d* = .29] and CP [*t*(190) = 2.29, *p* = .02, and *d* = .33], but not for *E*% [*t*(190) = .29, *p* = .77, and *d* = .06].

## 5. Discussion

The aim of this study was to examine the effect of an existing school-based PA opportunity, delivered during physical education lessons, on students' selective attention in the classroom. The unique contribution of this study lies on the fact that the intervention involved a regular physical education class (without changing the structure of the school day or the lesson), which demonstrates more contextually relevant ecological validity. Further, the form of PA used during the physical education lessons in this study included aerobic dance activities with unique qualitative characteristics (e.g., coordination and high cognitive engagement) compared to more common types of PA used in previous research, such as running (e.g., [19]).

The findings of this study suggest that the acute bouts of PA during physical education in the form of aerobic dance significantly improved Year 5 and 6 students' processing speed and concentration performance but not accuracy (i.e., percentage of errors). The effect sizes in the current study suggest a small to medium effect for the two measures that demonstrated significant improvement. When considering the findings of this study, it is important to remember that the intervention involved a 45-minute physical education lesson, in which students engaged in about 9 minutes of MVPA.

In discussing the findings of studies examining the effects of PA participation on cognitive performance, it is important to consider both quantitative and qualitative characteristics of PA. Given the nature of the activity used in this study (i.e., aerobic dance with coordinative and cognitive demands), it is useful to turn to the literature focused on interventions of various levels of cognitive engagement in acute PA sessions for comparisons. Cognitive engagement has been proposed as a potential psychological mechanism responsible for changes in executive functions following acute bouts of PA [5, 16]. Specifically, it is thought that cognitively engaging or challenging PA helps activate the prefrontal cortex, which in turn impacts executive functions [16]. Indeed, studies examining neural correlates of motor behavior suggest that the neural regions recruited during performance in motor tasks are the same as those associated with cognitive operations and that complex motor tasks are valuable in examining links between action and cognition [29].

Some of the studies in this area focused specifically on selective attention and used various interventions of cognitively engaging PA, including physical education and classroom contexts. For example, Schmidt et al. [30], who compared the effects of a 45-minute cognitively demanding physical education session (i.e., using coordinative exercises) and a normal sedentary school lesson, found improvements in focused attention and processing speed (but not accuracy) over time for both groups but no significant immediate intervention effect. Gallotta et al. [31] examined three conditions, involving cognitive exertion (school curricular lesson), physical exertion (traditional 50-minute physical education lesson), and mixed cognitive and physical exertion (coordinative 50-minute physical education lesson), and found an improvement in focused attention and processing speed over time in all conditions. However, they also found that the children in the mixed cognitive and physical exertion group improved less between pre- and postintervention compared to the other two groups. On the contrary, Budde et al. [20] found a significantly larger improvement in processing speed, accuracy, and concentration for students who engaged in a 10-minute session of coordinative exercise compared to students who engaged in a 10-minute normal sport lesson (low coordinative demands). Similarly, Schmidt et al. [13] compared four different 10-minute sessions of various physical and cognitive demands in the classroom and found no effect of physical exertion on students' attentional performance but a significant effect of cognitive engagement on focused attention and enhanced processing speed (but not accuracy).

Other studies in this area explored the effects of different cognitively engaging PA sessions on other measures of executive functioning. For example, Benzing et al. [32] found that an acute 15-minute cognitively engaging exergame-based PA session enhanced male adolescents' cognitive flexibility. Similarly, the results of Jäger et al. [21] indicated a significantly stronger improvement in inhibition, but not in updating and shifting, for the experimental group, who participated in a 20-minute cognitively engaging sport sequence (compared to a resting control condition). However, Jäger et al. [22], who examined the effects of qualitative different 20-minute acute PA interventions on the executive functions of 10–12-year-old children in real-world settings, did not find any significant effects of the conditions with and without cognitive engagement on executive functions in the overall sample.

Studies in this area employed different designs, populations, measures, and other characteristics, which makes comparisons among them and interpretation of conflicting findings challenging. However, one element that has received attention in this area is the duration of cognitively engaging acute PA sessions. Schmidt et al. [13] observed that no interventions lasting longer than 15 minutes demonstrated positive effects for cognitively engaging conditions and using the strength model of self-control [33] attributed this to a potential depletion of the limited capacity of self-control resources [34] after long sessions of cognitively demanding PA. This conflicts with the results of the current study, where students participated in a physical education session longer than 15 minutes and improvements in processing speed and concentration performance were found.

However, there are some important differences between this study and most other studies in this area that should be taken into consideration when attempting to interpret the findings. First, the intensity of PA in this study was not maintained at a moderate-to-vigorous level throughout the PA session; rather, the students were allowed to self-select the level of intensity. Indeed, maintaining a specific intensity level may be challenging to achieve in an authentic physical education lesson that involves behavioral and academic objectives, various management tasks, and possibly longer instructional episodes than interventions used in studies in this area. Thus, the difference in intensity between this study and previous studies that examined cognitively engaging conditions of durations longer than 15 minutes may be the reason underlying the conflicting findings. The lower intensity across the PA session in this study may have prevented the depletion of the available common capacity-limited reservoir of voluntary attention or mental effort [34].

Another difference relates to the type of PA used in this study (dance), which is substantially different than the ones used in previous studies and may have had implications for student enjoyment (which we did not measure in this study). As Audiffren and André [34] propose in their paper revisiting the strength model of self-control, improvements of performance in self-regulation tasks observed after acute exercise may indeed be explained by an increase in positive mood. Finally, while this study used an acute bout of dance as the intervention, students were exposed to dance as part of their regular physical education lessons, which may have implications for the level of cognitive engagement and, in turn, the self-control resources used during the session. Although it is difficult to explain the differences between the results of this study and other similar studies, the aforementioned factors, including duration, intensity, and levels of cognitive and emotional engagement, need to be considered in future studies in this area.

Despite differences in designs, participants, and other characteristics, several studies in this area focused on selective attention, which allows for some comparisons to be made. Comparably to the current study, the results of some relevant studies also indicate improvements in processing speed and concentration performance but not accuracy, as a result of participation in various acute bouts of cognitive demanding PA (e.g., [13, 30, 31, 35]). At the same time, contrary to the results of this study, Budde et al. [20] found improvement in all three measures of selective attention (including accuracy) after participation in an acute bout of coordinative exercise.

A potential explanation for the conflicting findings, which has also been discussed by Schmidt et al. [13, 30], may relate to the age of the participants. The participants in the study of Budde et al. [20] were 13–16 years old, whereas the age of the participants in the remaining studies ranged between 8–13 years. Indeed, according to cognitive developmental research, older children seem to gradually start favoring accuracy over speed compared to children of younger ages [36], which may explain the discrepancies in the findings of the studies discussed in this section. Another potential explanation for the lack of improvement in accuracy that has been proposed by Schmidt et al. [30] may be a lack of positive physiological influence and particularly catecholamine and glucocorticoid levels, due to insufficient physical exertion in the PA session. This may be the case in this study given that students in the intervention group accumulated about 9 minutes of MVPA during the physical education lesson.

Beyond the impact of the acute PA bout of dance on student's selective attention, a noteworthy finding in this study is the low levels of PA accumulated during the physical education lesson. Specifically, students in this study spent about 20% of the total scheduled lesson time (i.e., 45 minutes) in MPVA and accumulated less than 2000 steps, which are significantly lower than available recommendations (i.e., 50% of physical education lesson time spent in MVPA-IOM, 2013; 2000 steps in a 30-minute lesson [37]). This may be related to the fact that classroom teachers were the instructors for physical education in this primary school, which is common in NZ. While classroom teachers are capable of effectively leading PA opportunities, the literature suggests that they often lack the content knowledge, confidence, and competence in this area [38, 39], therefore highlighting the importance of having physical education specialists teaching physical education lessons. The individuals leading or facilitating PA opportunities may have an impact on the amount and quality of PA students accumulate but, perhaps more importantly, can also play a critical role in motivating students to participate in activities that can help improve their executive functioning [40].

This study is not without limitations. It was conducted in a single school in a high socioeconomic area, which limits the generalization of its findings to other settings. In addition, while the PA completed during the physical education lessons originated from the same program, some of those lessons were based on a video while some were facilitated by student leaders. Although both are common and recommended practices in the JUMP JAM program, they may have influenced student engagement in the intervention. Further, students' perceived cognitive engagement during the physical education lessons was not monitored. At the same time, the strengths of this study include the authentic nature of the use of an alternative activity in the physical education lessons and the use of a practical instrument (the d2 Test of Attention) that produces valid and reliable data (Brickenkamp, 2000).

Future research in this area should be conducted in authentic school-based contexts and employ a more diverse range of conditions, manipulating both quantitative (e.g., duration, intensity) and qualitative characteristics of PA sessions. Qualitative characteristics manipulated may involve different types of activities and curricula used during physical education lessons or different individuals leading/facilitating PA sessions. These characteristics can have implications for the levels of cognitive engagement, behavioral engagement (involvement in learning and academic tasks, such as effort, persistence), and emotional engagement (affective reactions such as interest, boredom, happiness, and anxiety) in PA sessions. While behavioral and emotional engagement have thus far not been explored much [13], they relate to children's motivation to engage and devote effort in an activity and are thus key elements of success in interventions/activities aiming to enhance executive function (e.g., [40–42]). Finally, future studies could also include a control condition that involves a mental, rather than a physical, break from academic work to help examine further whether PA participation (as opposed to simply taking a break from academic work) impacts executive functioning.

## 6. Conclusions

The findings of this study suggest that an acute bout of aerobic dance delivered during a regular physical education lesson, during which students engaged in about 9 minutes of MVPA, significantly improved students' processing speed and concentration performance, but not accuracy, in a selective attention test. This study extends the body of literature demonstrating significant improvements in various aspects of selective attention as a result of participation in acute bouts of cognitively engaging PA. Its findings suggest that movement and learning are not necessarily antagonists [14] and can help reconcile the educational and public health agendas imposed on schools, thus contributing to the continued and expanded offering of school PA opportunities. Finally, these findings point to the need to consider both quantitative and qualitative characteristics of PA when examining effects on cognitive performance and when planning PA sessions during the school day.

## Figures and Tables

**Table 1:** Results of the 2 (pretest, posttest) × 2 (comparison, intervention) ANOVA tests.

Measure	Time				Group				Group × time			
	*F*	df	*p*	Partial *η*^2^	*F*	df	*p*	Partial *η*^2^	*F*	df	*p*	Partial *η*^2^
TN	375.09	(1, 190)	*<*.001	.66	.04	(1, 190)	.85		4.12	(1, 190)	.03	.02
*E*%	67.88	(1, 190)	*<*.001	.26	.35	(1, 190)	.56		.18	(1, 190)	.77	
CP	500.15	(1, 190)	*<*.001	.73	.31	(1, 190)	.58		5.22	(1, 190)	.02	.03

*Note*. TN = total number of items processed; *E*% = percentage of errors; CP = concentration performance.

**Table 2:** Descriptive statistics for d2 Test of Attention components and physical activity.

	Mean	SD		Mean	SD	Difference *M*	Difference SD
Comparison							
Pretest TN	317.38	63.61	Posttest TN	363.94	72.61	46.57^*∗*^	34.27
Pretest *E*%	4.43	3.87	Posttest *E*%	2.71	3.59	−1.72	2.74
Pretest CP	121.31	27.13	Posttest CP	144.38	30.54	23.07^*∗*^	14.28
Intervention							
Pretest TN	310.15	56.91	Posttest TN	367.61	74.13	57.47^*∗*^	39.13
Pretest *E*%	4.79	4.43	Posttest *E*%	2.94	3.15	−1.85	3.27
Pretest CP	116.41	25.58	Posttest CP	144.73	33.43	28.32^*∗*^	17.56
			Steps	1931.16	421.41		
			MVPA (min:sec)	08:81	02:22		

*Note.* TN = total number of items processed; *E*% = percentage of errors; CP = concentration performance. ^*∗*^*p* < .05 for follow-up independent *t*-tests using difference scores.
